# Evaluation of Septoria Nodorum Blotch (SNB) Resistance in Glumes of Wheat (*Triticum aestivum* L.) and the Genetic Relationship With Foliar Disease Response

**DOI:** 10.3389/fgene.2021.681768

**Published:** 2021-06-29

**Authors:** Michael G. Francki, Esther Walker, Christopher J. McMullan, W. George Morris

**Affiliations:** ^1^Department of Primary Industries and Regional Development, South Perth, WA, Australia; ^2^State Agricultural Biotechnology Centre, Murdoch University, Murdoch, WA, Australia

**Keywords:** septoria nodorum, parastagonospora nodorum, glume, foliar, wheat, QTL, environment

## Abstract

Septoria nodorum blotch (SNB) is a necrotrophic disease of wheat prominent in some parts of the world, including Western Australia (WA) causing significant losses in grain yield. The genetic mechanisms for resistance are complex involving multiple quantitative trait loci. In order to decipher comparable or independent regulation, this study identified the genetic control for glume compared to foliar resistance across four environments in WA against 37 different isolates. High proportion of the phenotypic variation across environments was contributed by genotype (84.0% for glume response and 82.7% for foliar response) with genotype-by-environment interactions accounting for a proportion of the variation for both glume and foliar response (14.7 and 16.2%, respectively). Despite high phenotypic correlation across environments, most of the eight and 14 QTL detected for glume and foliar resistance using genome wide association analysis (GWAS), respectively, were identified as environment-specific. QTL for glume and foliar resistance neither co-located nor were in LD in any particular environment indicating autonomous genetic mechanisms control SNB response in adult plants, regulated by independent biological mechanisms and influenced by significant genotype-by- environment interactions. Known *Snn* and *Tsn* loci and QTL were compared with 22 environment-specific QTL. None of the eight QTL for glume or the 14 for foliar response were co-located or in linkage disequilibrium with *Snn* and only one foliar QTL was in LD with *Tsn* loci on the physical map. Therefore, glume and foliar response to SNB in wheat is regulated by multiple environment-specific loci which function independently, with limited influence of known NE-*Snn* interactions for disease progression in Western Australian environments. Breeding for stable resistance would consequently rely on recurrent phenotypic selection to capture and retain favorable alleles for both glume and foliar resistance relevant to a particular environment.

## Introduction

*Parastagonospora* (syn. ana, *Stagonospora*; teleo, *Phaeosphaeria*) *nodorum* (Berk.) Quaedvlieg, Verkley & Crous is the causal pathogen of Septoria nodorum blotch (SNB) of wheat that infects the lower leaves of the canopy and is identified by dark brown round or lens shaped spots that coalesce and develop black pycnidia as lesions mature (Eyal et al., 1987). Early foliar symptoms in Western Australia (WA) are seen at tillering (Feekes 5) and is a precursor to glume infection. Rain splash disperses spores whereby foliar disease symptoms proliferate under high humidity and infection continues up the canopy through to stem elongation and ripening. Infected heads will turn dark brown often with a purple tint and black pycnidia evident as typical glume blotch symptoms (Eyal et al., 1987). Yield losses are estimated to be approximately 12% where SNB is considered to be a major necrotrophic disease affecting grain yield in Western Australian production environments (Murray and Brennan, 2009) as well as other regions of the world, particularly as a recurrent disease of wheat in several geographical areas of the United States (Cowger et al., 2020). Fungicide applications provide opportunities for controlling the pathogen, but the use of SNB resistant cultivars can significantly reduce on-farm costs. However, breeding for leaf and glume blotch resistance is challenging due to the inherent genetic complexity controlling SNB response when the disease is most damaging (reviewed in Francki, 2013).

Similar to leaf blotch, glume blotch response is under quantitative control having additive-dominance effects for resistance with some interactions with non-allelic genes (Wicki et al., 1999). Dominance for glume blotch susceptibility is common (Fried and Meister, 1987; Wicki et al., 1999) whereas dominance for resistance also exists in specific crosses (Wicki et al., 1999). Moreover, morphological characteristics can have a profound effect on disease response so it is important to discriminate between pleiotropy and linkage with resistance in genetic analysis (Francki, 2013). There have been at least 20 QTL associated with glume resistance identified across the wheat genome with each accounting for up to 24% of the phenotypic variation indicating varying effects of each QTL contributing to resistance (Schnurbusch et al., 2003; Uphaus et al., 2007; Shankar et al., 2008; Czembor et al., 2019; Lin et al., 2020). Similarly, at least 18 QTL have been identified for foliar resistance (reviewed in Francki, 2013; Ruud and Lillemo, 2018) with subsequent reports of others that may represent existing or, indeed, new QTL (Czembor et al., 2019; Ruud et al., 2019; Francki et al., 2020; Lin et al., 2020). Despite the large number of loci, only few were detected in more than one environment including one QTL on 2D (Uphaus et al., 2007), 3A, 3B (Schnurbusch et al., 2003) and 4B (Schnurbusch et al., 2003; Shankar et al., 2008) for glume resistance and 1B (Francki et al., 2011); 7D (Czembor et al., 2019) and two on 2A (Lin et al., 2020) for foliar response. Many QTL detected for glume and foliar SNB response, therefore, appear to be environment-specific. Quantitative genetic analysis detected QTL for either glume or foliar SNB response in different field environments whereby some shared the same marker interval (Schnurbusch et al., 2003; Lin et al., 2020) indicated similar genes may have an effect on disease resistance or susceptibility in both organs. On the contrary, some studies did not detect the same QTL for glume and foliar resistance (Shankar et al., 2008; Czembor et al., 2019) confirming that alternative genes are seemingly under independent control and in agreement with earlier studies (Fried and Meister, 1987; Wicki et al., 1999). However, comparison between the genetic control of glume and foliar response to SNB in those studies were based on bi- (Shankar et al., 2008; Czembor et al., 2019) or multi-parental (Lin et al., 2020) populations where diversity is limited and the extent of alleles and effects on either resistance, susceptibility or both is not broadly exploited in global germplasm pools. Evaluation of a wider gene pool coupled with high marker density genetic mapping would further extrapolate allelic diversity and gene interactions to expand our knowledge on similar and/or independent genetic mechanisms controlling both glume and foliar SNB response in WA environments.

*P. nodorum* expresses a range of necrotrophic effectors (NE) that interact with corresponding sensitivity loci (*Snn*) that induce necrosis in wheat (reviewed in McDonald and Solomon, 2018). There were nine NE-*Snn* interactions identified in wheat on chromosomes 1A, 1B, 2A, 4B, 5B, and 6A (Abeysekara et al., 2009; Friesen et al., 2009, 2012; Gao et al., 2015; Shi et al., 2015; Phan et al., 2016; Ruud et al., 2017; Downie et al., 2018). It was shown that some *Snn* loci may play a role in foliar disease progression under SNB infection in multiple field environments (Friesen et al., 2009) while other studies indicated known NE-*Snn* interactions were either inconsistent or not associated with QTL in controlling disease development in different environments when inoculated with single or a mixture of isolates (Ruud and Lillemo, 2018; Czembor et al., 2019; Ruud et al., 2019; Francki et al., 2020; Lin et al., 2020). Interestingly, it has been suggested that known NE-*Snn* interactions are not a significant determinant for foliar response in eastern soft red winter wheat germplasm but the effect of unknown *Snn* loci cannot be excluded (Cowger et al., 2020). Similar observations and conclusions were drawn when an extensive collection of wheat germplasm from different regions of the world were evaluated in multi-environments using mixture of isolates from Western Australia (Francki et al., 2020). Despite the increased knowledge of NE-*Snn* interactions controlling foliar response to SNB, the role of characterized NE-*Snn* interactions for glume susceptibility and resistance is largely unknown in WA environments.

Genome-wide association studies (GWAS) provide an opportunity to simultaneously evaluate wheat accessions and identify the genetic basis of trait variation through marker-trait associations (MTA). GWAS is used increasingly to identify the genetic control of foliar response to SNB using germplasm representing a wider representation of alleles from different regions of the world (Ruud et al., 2019; Francki et al., 2020). High-density single nucleotide polymorphic (SNP) markers using the iSelect Infinium 90K SNP genotyping array (Wang et al., 2014) have provided a finer resolution of QTL and their association with previous known genetic and *Snn* specific loci. In multi-environment analysis, QTL for foliar SNB response were detected as environment specific on chromosomes 1A, 1B, 4B, 5A, 5B, 6A, 7A, 7B, 7D (Francki et al., 2020) whereas loci on chromosomes 2B, 2D, 4A, 4B, 5A, 5B, 6B, 7A, and 7B were detected in more than one field environment (Ruud et al., 2019) but with no common QTL confirmed between studies. The relationship between NE-*Snn* interactions and foliar disease response in adult plant in GWAS was largely inconsistent across multiple environments (Ruud et al., 2019; Cowger et al., 2020; Francki et al., 2020). To date, GWAS has neither been applied to investigate the genetic control for glume blotch resistance nor its association with known NE-*Snn* loci from a wider representation of alleles in global germplasm. Finer mapping resolution using GWAS and the iSelect Infinium 90K SNP genotyping array (Wang et al., 2014) will provide an in-depth analysis and increase our knowledge on the relationship between glume and foliar response and known NE-*Snn* interactions when adult plants are infected with different isolates across multiple field environments in WA.

Although consistent and high disease pressure enabled a reliable evaluation of foliar resistance to SNB across six WA environments (Francki et al., 2020), the lack of sustained disease progression during the grain filling period at five sites in 2016–2018 precluded reliable analysis for glume response. The aim of this study, therefore, was to evaluate glume response to SNB for 232 wheat lines in successive year field trials in environments where sustained glume blotch disease progression was consistent during the grain fill period in Western Australia. Moreover, the study aimed to identify genotype-by-environment interactions, compare and contrast the genetic control of glume with foliar response using GWAS and ascertain the significance of NE-*Snn* interactions in WA environments. The outcome of the study will provide knowledge on shared or independent genetic determinants regulating glume and foliar resistance to SNB in global wheat germplasm when evaluated in multiple field environments under different isolates.

## Materials and Methods

### Plant Material

The GWAS panel consisted of 232 hexaploid wheat lines including 71 lines from Australia, 72 inbred and commercial lines from Centro Internacional de Mejoramiento de Maiz y Trigo (CIMMYT), 78 inbred lines from International Center for Agricultural Research in the Dry Areas (ICARDA), and 11 landraces from various origins. Description of lines, pedigrees and their origins for the GWAS population used in this study was reported in Francki et al. (2020) and detailed in Supplementary Table 1.

### Field Trial Design

Trials were sown at Department of Primary Industries and Regional Development (DPIRD) Manjimup Research Station and DPIRD South Perth Nursery (Western Australia) in 2018–2020 and 2020, respectively. All trials were sown as completely randomized designs with three replications for each genotype. Plots in each trial at Manjimup were sown as two-rows of 1.9 m length and 0.2 m row spacing. Each row contained ∼100 seeds. The susceptible cultivar “Amery,” a Western Australian variety with consistent SNB susceptibility across environments (Francki et al., 2020), was sown as two-row plots of 1.9 m length adjacent to each treatment plot to maintain consistency in disease progression. In the 2020 South Perth trial, plots were sown as two-rows of 0.5 m length and 0.2 m spacing with a spreader two-row plot (“Amery”) of 0.5 m length adjacent to each treatment. Each row contained ∼25 seeds. The susceptible genotypes for glume and leaf blotch (three replications) included “Millewa,” “Arrino,” “Scout” and the landrace, 040HAT10, were sown in each trial at Manjimup and South Perth and used to monitor disease progression. EGA Blanco, 30ZJN09 and 6HRWSN125 were sown as resistant check genotypes.

### Isolates, Culture Preparation and Inoculation of Field Trials

Isolates of *P. nodorum* were sourced from the culture collection at DPIRD and collected from different regions of WA. A total of 19, 17 and 12 isolates were selected and used in equal amounts as mixed inoculum for trials in 2018, 2019, and 2020, respectively (Supplementary Table 2). At least 40% of the isolates used in each year were represented in the mixed inoculum in the following year with three common isolates, WAC13077, WAC13206 and WAC13872 used in all trials (Supplementary Table 2). Fungal cultures and mixed inoculum consisting of equal amounts of *P. nodorum* isolates (10^6^ spores/ml) were prepared and all plots for each trial were inoculated at a rate of 28.5 m^2^/L as previously described (Francki et al., 2020). Inoculation in each trial commenced from formed tillers to leaf sheaths lengthening and strongly erect (Feekes 3–5) with three subsequent inoculations at 14-day intervals.

### Environment Characterization, SNB Disease and Agronomic Measurements

Trials at DPIRD Manjimup research station and South Perth nursery were in close proximity to weather stations for recording of climatic conditions including air temperature, relative humidity, rainfall, solar exposure and pan evaporation to identify parameters that may affect disease progression within and between environments. Climate data was recorded daily and accessed through DPIRD weather and radar database.^1^ Thermal time (^o^Cd) for the duration of disease progression was calculated using the sum of average daily minimum and maximum air temperature as ∑(*min*⁡*temp*+*max*⁡*temp*/2) from the day of first inoculation to the day of disease measurement.

Susceptible check varieties were monitored weekly for SNB disease progression and visually assessed for necrosis on the glume and flag leaf and recorded on a percent glume area disease (PGAD) and percent flag-leaf area disease (PLAD) scale as described by James (1971). Trials were visually assessed for other necrotrophic diseases weekly, particularly yellow spot, with no symptoms detected in any of the trials. Each plot scored five individual random plants for SNB disease symptoms from the middle of the row closest to the spreader susceptible plot. PLAD on the flag leaf represented foliar disease whereas PGAD on the head represented glume disease for each replicate. All plots in the trial were scored at the same time when at least two check susceptible varieties had PGAD > 50% and PLAD > 70%. Foliar and glume disease scores for each replicate determined mean plot values.

Heading date was measured from the number of days from sowing for each replicate to reach 50% full head emergence. Plant height was measured for three random plants from the middle of the row closest to the spreader susceptible plot. Height (cm) was taken from the soil level to the top of the head (excluding awns) and mean plot values was used for statistical analysis.

### Statistical Analysis

All statistical analyses for phenotypic evaluation were done using Genstat, 19th edition.^2^ Generalized linear models and linear mixed models were used in phenotypic analysis of trait data. Treatment factors, plant height and heading date used as co-variates were fitted to fixed models to estimate main effects and interactions. Best linear unbiased predictors (BLUP) for PGAD and PLAD were calculated for each environment based on linear mixed models assuming fixed effect for genotypes and used for subsequent QTL analysis using GWAS. Finlay-Wilkinson joint regression analysis was used to compare genotypes for SNB response and agronomic traits across four environments. Broad-sense heritability estimates were calculated using the formula *H*^2^ = σ_*g*_^2^/σ_*p*_^2^ where σ_*g*_^2^ and σ_*e*_^2^ are the genotypic and phenotypic variances, respectively (Wray and Visscher, 2008).

### Genome-Wide Association Analysis

As the same wheat lines in this study were used previously, detailed methodology for genotyping, analysis of population structure and genome wide association was previously described by Francki et al. (2020). Briefly, the 232 wheat lines were genotyped using the 90K Infinium SNP chip array (Wang et al., 2014) and SNP markers with <80% call rate and <5% minor allele frequencies were removed resulting in a total of 20,563 SNPs used for analysis. TASSEL v.5.2.52 was used to identify marker-trait associations (MTAs) (Bradbury et al., 2007). A mixed linear model (MLM) was determined to be the most appropriate to account for both structure and cryptic relatedness for this population (Francki et al., 2020). The genotypic kinship matrix (K) was estimated by selecting the “Centered_IBS” method and population structure (Q) was corrected using principal component (PC) analysis. The suitable number of PCs for each trait was determined by testing one through 15 PCs with visual assessment of quantile-quantile plots (Q-Q plots). The option “P3D” was not selected during the MLM analysis with the variance component re-estimated after each marker. The R programs “qqman” and “Rcolorbrewer” were used to draw Manhattan plots (Turner, 2017; R Core Team, 2018). A genome-wide significance threshold for MTAs was set at *p* < 2.43 × 10^–6^ (−log_10_ (*p*) > 5.61) using Bonferroni correction with α = 0.05. To estimate the number of independent tests the tagger function in Haploview was implemented as described in Maccaferri et al. (2016) with a *r*^2^ of ≤ 0.1. This returned a genome-wide moderate threshold significance of *p* < 7.65 × 10^–5^ (−log_10_ (*p*) > 4.12). A suggestive threshold of significance of *p* < 1 × 10^–3^(−log_10_ (*p*) > 3.00) was also included in Manhattan plots as previously reported (Gao et al., 2016; Alomari et al., 2017; Muqaddasi et al., 2019).

Marker pairwise *r*^2^ values were calculated in PLINK 1.9 (Purcell et al., 2007) with a sliding window of 50 and LD decay curves fitted by non-linear regression for each genome (A, B and D) as described by Marroni et al. (2011) with decay of *r*^2^ against distance. LD decay plots were drawn in R with a critical threshold of *r*^2^ = 0.2 (R Core Team, 2018). MTA for QTL were defined to be in LD when their physical distance was within the linkage decay value for their respective sub-genomes.

### Assignment of QTL, *Snn* and *Tsn1* to the Physical Map

Physical locations of SNP markers were obtained using Pretzel v2.2.6, an interactive, web-based platform for navigating multi-dimensional wheat datasets, including genetic maps and chromosome-scale physical assemblies (Keeble-Gagnère et al., 2019). *Snn* and *Tsn1* loci were anchored to the physical map using SNP markers, or the closest linked SSR markers, as described in Francki et al. (2020). For markers not available in Pretzel v2.2.6, putative locations were obtained using the IWGSC RefSeq v1.0 and the BLAST tool at URGI INRA.^3^

## Results

### Environment Characterization

Daily average climate measurements during disease progression at DPIRD Manjimup research station in 2018–2020 were consistent in successive years for air temperature, relative humidity, rainfall, solar exposure and pan evaporation (Supplementary Table 3). Similarly, the total rainfall recorded was 500, 411, and 441 mm in 2018, 2019, and 2020, respectively. The climatic conditions at Manjimup WA, therefore, were consistent in 2018–2020. However, the site at South Perth WA was higher in average daily air temperature, solar exposure and pan evaporation but lower for relative humidity and rainfall compared to any year at Manjimup (Supplementary Table 3), with considerably less total rainfall of 313 mm in the period from first inoculation to final disease score. The trial at South Perth in 2020, therefore, provided an opportunity to compare the response of 232 wheat lines to glume and foliar SNB infection under different climatic environments.

### Assessment of Glume Response to SNB

A total of 232 wheat lines were evaluated for glume and leaf response to SNB in each year at Manjimup (2018–2020) and at South Perth in 2020. Thermal times for disease evaluation when PGAD was >50% for at least two susceptible check varieties at Manjimup was 1117 ^o^Cd -1238^*o*^Cd in 2018–2020 but higher (1589^*o*^Cd) at South Perth (Supplementary Table 4) indicating climate differences affected rate of progression of glume blotch symptoms. Nevertheless, glume response showed consistently high heritability across all sites (*H*^2^ = 0.79 to 0.89; Table 1) indicating that a significant proportion of phenotypic difference within each environment is controlled by genetic variation. The mean and median of the population for glume response were similar (29.0 to 33.0 and 27.0 to 30.0, respectively; Table 1) indicating comparable disease pressure for glume response across environments within and between years and normal distribution of disease response for the 232 wheat lines (Supplementary Figure 1) in each environment. There was a high and significant linear relationship for PGAD scores between successive trials at Manjimup (*r* = 0.76 to 0.82; *P* < 0.001) and between the South Perth and Manjimup trials (*r* = 0.73 to 0.78; *P* < 0.001) indicating consistent glume response of genotypes across all environments (Table 2). There was moderate negative correlation between heading date and PGAD in each trial (*r* = −0.46 to −0.63; *P* < 0.001) and low to moderate negative correlation between plant height and PGAD (*r* = −0.34 to −0.64; *P* < 0.001) in each environment (Table 3) indicating potential pleiotropic effects between morphological traits and glume response. The genotype, environment and their interactions were fitted as terms in a linear mixed model and the significant proportion of glume response was attributed to genotype (84%) followed by genotype-by-environment interactions (14.7%) with only small proportion of the variation (1.3%) attributed by the environment (Table 4).

**TABLE 1:** Summary of percent glume area disease (PGAD), percent leaf area disease (PLAD), heading date (HD) and plant height (PH) for 232 global wheat lines evaluated in four environments in Western Australia in 2018–2020.

	**Manjimup 2018**				**Manjimup 2019**				**Manjimup 2020**				**South Perth 2020**			
	**PGAD**	**PLAD^a^**	**HD^a^**	**PH^a^**	**PGAD**	**PLAD**	**HD**	**PH**	**PGAD**	**PLAD**	**HD**	**PH**	**PGAD**	**PLAD**	**HD**	**PH**
Minimum	4.0	2.0	86.0	73.0	1.0	4.0	89.0	71.0	0.0	4.0	95.0	63.0	3.0	3.0	81.0	68.0
Maximum	78.0	97.0	133.0	123.0	83.0	90.0	126.0	117.0	72.0	95.0	131.0	117.0	75.0	97.0	113.0	107.0
Grand Mean	29.0	38.0	111.0	94.0	29.0	40.0	109.0	94.0	30.0	44.0	113.0	89.0	33.0	43.0	93.0	83.0
Median	27.0	36.0	110.0	94.0	27.0	38.0	108.0	93.0	30.0	43.0	113.0	89.0	30.0	42.0	92.0	82.0
Mode	17.0	53.0	115.0	94.0	45.0	52.0	106.0	94.0	45.0	60.0	117.0	91.0	53.0	50.0	89.0	82.0
ANOVA (*P*)	*P*<0.001	*P*<0.001	*P*<0.001	*P*<0.001	< 0.001	< 0.001	< 0.001	< 0.001	< 0.001	< 0.001	< 0.001	< 0.001	< 0.001	< 0.001	< 0.001	< 0.001
LSD (*P*<0.05)	15.3	19.6	4.9	6.8	14.8	24.4	4.2	6.2	15.3	21.9	5.7	8.6	17.9	21.8	6.6	6.9
CV (%)	31.7	30.6	2.8	4.5	32.4	15.6	2.4	4.2	31.5	31.3	3.1	6.0	34.1	31.5	4.4	5.2
*H*^2^	0.79	0.88	0.94	0.90	0.89	0.91	0.92	0.96	0.86	0.79	0.88	0.78	0.86	0.89	0.81	0.80

*^*a*^PLAD and agronomic scores reported in Francki et al. (2020).*

**TABLE 2:** Phenotypic correlation between four trials at Manjimup (MJ) and South Perth (SP) Western Australia in 2018–2020 of 232 wheat lines for percent glume and leaf area diseased (PGAD and PLAD, respectively).

	**PGAD**				**PLAD**			
	**MJ2018**	**MJ2019**	**MJ2020**	**SP2020**	**MJ2018**	**MJ2019**	**MJ2020**	**SP2020**
MJ2018	–	–	–	–	–	–	–	–
MJ2019	0.78**	–	–	–	0.82**	–	–	–
MJ2020	0.76**	0.82**	–	–	0.71**	0.75**	–	–
SP2020	0.73**	0.78**	0.75**	–	0.75**	0.77**	0.68**	–

**P<0.001.

**TABLE 3:** Phenotypic correlations (*r*) between percent glume and leaf area disease (PGAD and PLAD, respectively), heading date (HD) and plant height (PH) at four environments in Western Australia in 2018–2020.

	**Manjimup 2018**				**Manjimup 2019**				**Manjimup 2020**				**South Perth 2020**			
	**PGAD**	**PLAD**	**HD**	**PH**	**PGAD**	**PLAD**	**HD**	**PH**	**PGAD**	**PLAD**	**HD**	**PH**	**PGAD**	**PLAD**	**HD**	**PH**
HD	−0.53**	−0.66**			−0.58**	−0.70**	–		−0.46**	−0.54**	–		−0.63**	−0.59**	–	
PH	−0.64**	−0.59**	0.40**		−0.48**	−0.52**	0.33**	–	−0.34**	−0.40**	0.37**	–	−0.45**	−0.37**	0.21*	–

**P < 0.001; *P < 0.01.

**TABLE 4:** Linear mixed model analysis for genotypes, environments and their interactions with respect to percent glume and leaf area diseased (PGAD and PLAD, respectively) for 232 wheat lines evaluated in four environments in Western Australia in 2018–2020.

	**PGAD**				**PLAD**			
**Source of variation**	**Wald statistic**	***F***	***P*^a^**	**%Var^b^**	**Wald statistic**	***F***	***P*^a^**	**%Var^b^**
Genotype (G)	6189.21	19.46	< 0.001	84.0	6245.22	19.04	< 0.001	82.7
Environment (E)	96.83	32.28	< 0.001	1.3	80.37	26.79	< 0.001	1.1
GxE	1079.16	1.77	< 0.001	14.7	1222.59	2.04	< 0.001	16.2

*^*a*^*F*-test probability of Wald statistic. ^*b*^Percentage of variation associated with each term or interaction.*

### Assessment of Foliar Response to SNB

Similar to glume response, thermal times (when PLAD was >70% for at least two susceptible check varieties) were comparable between years at Manjimup but lower than at South Perth (Supplementary Table 4), indicating climate affected rate of foliar disease progression between geographical locations. PLAD on flag leaves representing foliar response to SNB showed consistently high broad-sense heritability (*H*^2^ = 0.79–0.91) and comparable population mean, median and mode (Table 1) with either normal or edge-peaked distribution for foliar disease response (Supplementary Figure 1) between environments. High Pearson’s correlation was evident (*r* = 0.68 to 0.82; *P* < 0.001) indicating comparable foliar response of genotypes across four environments (Table 2). As with glume response, a moderate but significant negative correlation was observed between foliar response and morphological traits including heading date (*r* = −0.54 to −0.70; P < 0.001) and plant height (*r* = −0.37 to −0.59; *P* < 0.001) (Table 3). The phenotypic variation for foliar response contributed by genotype, environment and their interactions was similar to glume response with genotype and genotype-by environment interactions accounting for most of the variation (82.7 and 16.2%, respectively) while environmental effects (1.1%) contributed the smallest proportion of variation across environments (Table 4).

### Comparison of Glume and Foliar Response to SNB

The moderate to high Pearson’s correlation (*r* = 0.56 to 0.82; *P* < 0.001) observed between PGAD and PLAD (Table 5) indicated a higher proportion of wheat lines have similar SNB response for glume and foliar disease when evaluated in a given environment regardless of the same or different isolates used as inoculum. A Finlay and Wilkinson joint regression model identified 35 lines as glume resistant (PGAD < 20%) across four environments in 2018–2020 ranked in ascending order based on sensitivity to SNB response compared to susceptible control lines with similar heading date and plant height (Table 6). Furthermore, 21 lines identified as resistant to glume infection also had moderate resistance to foliar disease with PLAD < 30% (Table 6). The remaining 14 lines identified as glume resistant were identified as moderately susceptible or susceptible to foliar disease (PLAD > 30%) similar to the susceptible control lines (Table 6). Therefore, similarities and differences in glume and foliar SNB response of individual genotypes indicated that either comparable or alternative genetic loci play a role in controlling resistance and susceptibility in different organs of adult plants when evaluated across multiple WA environments.

**TABLE 5:** Pearson’s correlation coefficient between four trials at Manjimup (MJ) and South Perth (SP) Western Australia in 2018–2020 of 232 wheat lines for percent glume and leaf area diseased (PGAD and PLAD, respectively).

	**PGAD MJ2018**	**PGAD MJ2019**	**PGAD MJ2020**	**PGAD SP2020**	**PLAD MJ2018**	**PLAD MJ2019**	**PLAD MJ2020**	**PLAD SP2020**
PGAD MJ2018	–							
PGAD MJ2019	0.78**	–						
PGAD MJ2020	0.76**	0.82**	–					
PGAD SP2020	0.73**	0.78**	0.75**	–				
PLAD MJ2018	0.81**	0.67**	0.65**	0.62**	–			
PLAD MJ2019	0.73**	0.82**	0.71**	0.66**	0.82**	–		
PLAD MJ2020	0.63**	0.66**	0.76**	0.56**	0.71**	0.76**	–	
PLAD SP2020	0.65**	0.68**	0.63**	0.71**	0.75**	0.77**	0.68**	–

**P < 0.001.

**TABLE 6:** Assessment of wheat lines using Finlay-Wilkinson joint regression for low mean PGAD scores (<20%) and stability across four WA environments with corresponding PLAD scores, heading date and plant height compared with control susceptible lines in 2018–2020.

	**PGAD**			**PLAD**			**Heading Date**	**Plant Height**
**Varieties/Inbreds**	**Mean (s.e.)**	**Sensitivity (s.e.)^*a*^**	**Mean square deviation^*b*^**	**Mean (s.e.)**	**Sensitivity (s.e.)**	**Mean square deviation**	**Mean (s.e.)**	**Mean (s.e.)**
**PGAD resistant**								
EGA Bonnie Rock	9.95 (9.7)	−17.33 (7.3)	89.3	35.42 (6.8)	−4.78 (4.1)	68.2	104.3 (2.9)	91.4 (2.5)
ZWW09Qno177	13.11 (9.4)	−12.53 (6.2)	210.0	49.02 (6.8)	−5.07 (2.9)	171.7	103.1 (2.8)	90.7 (2.5)
EGA Blanco	6.01 (9.7)	−3.75 (7.3)	32.8	11.60 (6.8)*	−4.25 (4.0)	43.9	108.6 (2.9)	92.1 (2.5)
53:ZIZ12	15.30 (9.4)	−3.75 (6.2)	212.8	34.83 (6.8)	−1.60 (3.0)	261.6	111.7 (2.8)	96.9 (2.5)
ZEE10Qno133	13.62 (9.4)	−3.68 (6.2)	192.6	41.8 (6.8)	1.96 (2.9)	60.2	107.7 (2.8)	84.8 (2.5)
ZVS07Qno227	7.44 (9.4)	−2.94 (6.2)	19.2	41.44 (6.8)	5.70 (2.9)	208.3	104.5 (2.8)	94.7 (2.5)
ZWW09Qno72	16.22 (9.4)	−2.94 (6.2)	58.0	48.2 (6.8)	0.89 (2.9)	83.5	111.2 (2.8)	91.5 (2.5)
ZWB11Qno95	3.88 (9.4)	−2.15 (6.2)	13.4	26.49 (6.8)	5.27 (2.9)	56.5	110.4 (2.8)	91.3 (2.5)
WAWHT2046	16.67 (3.0)	−1.77 (1.2)	71.1	15.33 (3.8)*	−1.57 (1.1)	85.2	103.6 (1.0)	96.7 (1.4)
ZWW10Qno139	6.91 (9.4)	−1.25 (6.2)	64.8	10.99 (6.8)	−1.26 (2.9)	69.9	110.0 (2.5)	100.5 (2.5)
ZEE10Qno77	9.78 (11.3)	−0.23 (7.2)	24.1	24.49 (8.2)*	5.40 (3.3)	34.4	109.8 (2.9)	100.4 (3.0)
ZWW10Qno60	13.51 (9.4)	−0.19 (6.2)	23.9	40.78 (6.8)	−0.25 (2.9)	262.9	111.4 (2.8)	89.3 (2.5)
Pfau	14.83 (3.0)	−0.09 (1.2)	63.5	22.68 (3.8)	−1.51 (1.1)	69.3	110.4 (1.0)	90.8 (1.4)
Yandanooka	17.5 (3.0)	0.11 (1.2)	52.4	37.00 (3.8)	−2.67 (1.1)	77.8	108.2 (1.0)	95.6 (1.4)
54:ZIZ13	9.00 (3.0)	0.26 (1.2)	38.3	19.50 (3.8)	0.36 (1.1)	151.6	111.2 (1.0)	97.2 (1.4)
159:ZIZ13	19.67 (3.0)	0.30 (1.2)	50.0	36.67 (3.8)	1.27 (1.1)	81.8	111.6 (1.0)	91.7 (1.4)
75:ZIZ13	17.50 (3.0)	0.34 (1.2)	27.1	22.58 (3.8)	1.07 (1.1)	177.5	111.3 (1.0)	85.6 (1.4)
6HRWSN125	5.83 (3.0)	0.38 (1.2)	21.9	13.92 (3.8)*	−0.23 (1.1)	248.2	105.5 (1.0)	97.9 (1.4)
Brookton	14.92 (3.0)	0.47 (1.2)	13.1	27.02 (3.8)	3.59 (1.1)	202.2	109.8 (1.0)	94.2 (1.4)
Bumper	15.83 (3.0)	0.71 (1.2)	33.3	24.67 (3.8)	−2.31 (1.1)	162.5	107.8 (1.0)	94.9 (1.4)
Lang	16.67 (3.0)	0.73 (1.2)	86.0	26.17 (3.8)	1.18 (1.2)	132.8	109.7 (1.0)	90.1 (1.4)
ZJN10Qno12	11.08 (3.0)	0.92 (1.2)	55.1	16.62 (3.8)	1.78 (1.1)	82.7	109.9 (1.0)	99.2 (1.4)
88:ZIZ13	15.17 (3.0)	1.06 (1.2)	111.0	32.17 (3.8)	−0.00 (1.1)	60.0	109.9 (1.0)	89.7 (1.4)
110:ZIZ13	16.83 (3.0)	1.18 (1.2)	17.0	26.08 (3.8)	0.89 (1.1)	243.4	110.0 (1.0)	94.0 (1.4)
Excalibur	16.00 (3.0)	1.21 (1.2)	26.9	33.08 (3.8)	1.50 (1.1)	251.9	108.5 (1.0)	90.6 (1.4)
ZWW10Qno31	12.61 (9.4)	1.23 (6.2)	106.1	29.04 (6.8)	6.34 (3.0)	201.5	112.2 (2.8)	96.3 (2.5)
Sokoll	12.00 (3.0)	1.26 (1.2)	62.1	51.02 (3.8)	3.93 (1.1)	202.8	108.5 (1.0)	91.8 (1.4)
56:ZIZ13	18.33 (3.0)	1.32 (1.2)	38.0	36.08 (3.8)	−0.09 (1.1)	183.8	106.8 (1.0)	93.6 (1.4)
EGA Castle Rock	15.51 (4.5)	1.39 (1.4)	93.7	10.03 (5.5)*	−0.19 (1.3)	9.2	101.8 (1.6)	96.6 (2.0)
Suntop	17.58 (3.0)	1.45 (1.2)	70.5	28.05 (3.8)	3.78 (1.1)	352.1	111.0 (1.0)	91.1 (1.4)
30ZJN09	8.17 (3.0)	1.81 (1.2)	8.2	22.30 (3.8)*	−1.01 (1.1)	98.2	106.8 (1.0)	94.7 (1.4)
Tammin	13.33 (3.0)	2.88 (1.2)	48.3	12.85 (3.8)*	−0.544 (1.1)	61.9	112.2 (1.0)	89.9 (1.4)
Ajana	17.92 (3.0)	3.20 (1.2)	100.9	42.63 (3.8)*	4.77 (1.1)	219.9	106.3 (1.0)	90.4 (1.4)
ZWW09Qno157	19.93 (9.4)	4.79 (6.2)	13.5	27.81 (6.8)	3.22 (2.9)	29.3	110.4 (2.8)	102.6 (2.5)
ZVS09Qno133	18.64 (9.4)	5.85 (6.2)	19.1	16.38 (6.8)*	0.47 (2.9)	19.7	110.1 (2.8)	93.7 (2.5)
**PGAD susceptible**								
Millewa	60.83 (3.0)	1.88 (1.2)	113.1	81.25 (3.8)	−1.44 (1.1)	73.0	105.1 (1.0)	84.2 (1.4)
Arrino	36.92 (3.0)	2.69 (1.2)	86.8	52.08 (3.8)	1.70 (1.1)	88.6	101.8 (1.0)	86.5 (1.4)
Scout	31.08 (3.0)	2.95 (1.2)	157.2	47.92 (3.8)	3.31 (1.1)	116.4	111.6 (1.0)	86.4 (1.4)
040HAT10	47.52 (3.2)	3.30 (1.4)	84.0	59.86 (4.0)	1.21 (1.3)	294.0	107.7 (1.1)	91.0 (1.4)

*Wheat lines are ordered according to decreased sensitivity for glume SNB response. Standard error is denoted by s.e. Wheat lines with low PLAD scores evaluated in 2016–2018 (Francki et al., 2020) are shown with an asterisk. ^*a*^Genotypes ranked in ascending order from the most stable assessed by sensitivity of PLAD response to environmental effects. ^*b*^Predictability of response to SNB assessed by mean square deviation.*

### QTL for Glume and Foliar Response to SNB and Relationship With Known NE-*Snn* Interactions

The genetic relatedness of the GWAS panel was previously reported to have low population structure with 15.6% of the genetic variance accounted for in the first three principal components using the 20,563 filtered SNP markers (Francki et al., 2020). Linkage decay for physical distance was estimated by non-linear regression at 9.6 Mbp, 14.9 Mbp, and 21.0 Mbp for the A, B and D sub-genomes, respectively, for threshold *R*^2^ = 0.2 (Supplementary Figure 2). The linkage decay values were used as estimates for markers in LD when multiple significant MTA were identified in similar genomic regions on the physical map.

GWAS was used to identify shared and independent genomic regions that control glume and foliar response to SNB in different environments. Heading date and plant height were fitted as co-variates in a general linear model to reduce confounding pleiotropic effects of plant morphology on disease scores in each environment. BLUP for PGAD and PLAD were subsequently used for MTA in GWAS analysis. Q-Q plots showed deviations of the observed associations compared to the null hypothesis indicating SNP markers are associated with glume and foliar SNB response with QTL detected for at least a moderate level of significance of *p* < 7.65 × 10^–5^ (−log_10_ (*p*) > 4.12) in Manhattan plots for each environment (Supplementary Figures 3, 4). There were eight QTL detected on chromosomes 1D, 2A, 3A and 7B having at least moderate threshold significance of −log_10_ (*p*) ≥ 4.12 for glume response to SNB from four environments (Table 7). The estimated allelic effects ranged from 7.72 to 20.93% (Table 7) indicating the difference in average phenotypic values for each MTA between contrasting homozygous genotypes. Interestingly, only one region at 423.20 Mbp on chromosome 2A was associated with QTL in more than one environment (*QSng.MJ18.daw-2A.2* and QSng.MJ19.daw-2A) possibly representing a similar gene at this locus controlling glume response to SNB in two environments. The remaining were environment-specific as they neither co-located nor in LD with QTL for glume response detected from other sites (Table 7). QTL for heading date and plant height with small allelic effects (4.61–12.35% and 4.67–10.75%, respectively) were detected in some environments in 2018–2020 (Supplementary Table 5) but none were co-located or in LD with QTL for glume resistance (Table 7). Therefore, QTL for glume resistance was unlikely to be associated with morphological characteristics.

**TABLE 7:** Summary of marker trait associations for PGAD (glume) and PLAD (foliar) scores from four Western Australian environments in 2018–2020.

**Environment**	**Trait**	**QTL**	**Chromosome**	**SNP id**	**SNP name**	**SNP^*a*^**	**IWGSC-bp^*b*^**	**Consensus map position-cM^*c*^**	**R^2^**	**MAF^*d*^**	**Allele effect estimate %^*e*^**	***p*-value**	**–log_10_(*p*)**
Manjimup 2018	Glume	QSng.MJ18.daw-2A.1	2A	IWB59332	RAC875_c57998_165	[T/**C**]	202,872,663	341.14	0.08	0.42	20.25	3.56E-05	4.45
		QSng.MJ18.daw-2A.2	2A	IWB35263	IAAV6884	[**T**/C]	423,204,105	341.14	0.10	0.45	−20.93	2.18E-06	5.66
		QSng.MJ18.daw-2A.3	2A	IWB908	BobWhite_c1634_563	[A/**G**]	453,520,296	341.14	0.07	0.46	16.77	7.61E-05	4.12
			2A	IWB51426	Ra_c21219_505	[**A**/G]	461,417,569	348.36	0.08	0.45	−18.86	3.85E-05	4.41
Manjimup 2019	Glume	QSng.MJ19.daw-2A	2A	IWB35263	IAAV6884	[**T**/C]	423,204,105	341.14	0.09	0.45	−20.93	1.49E-05	4.83
Manjimup 2020	Glume	QSng.MJ20.daw-1D	1D	IWB35174	IAAV6247	[**A**/G]	10,661,637	53.03	0.08	0.41	−7.72	7.20E-05	4.14
			1D	IWB26984	Excalibur_c4876_832	[A/**G**]	10,662,717	53.03	0.10	0.43	8.31	4.50E-06	5.35
			1D	IWB18376	D_GBF1XID01C7T2Q_63	[T/**C**]	10,668,578	53.03	0.09	0.40	7.92	3.90E-05	4.41
			1D	IWA7533	wsnp_Ra_c1020_2062200	[**A**/G]	10,719,634	53.03	0.09	0.45	−7.75	2.62E-05	4.58
South Perth 2020	Glume	QSng.SP20.daw-1D	1D	IWB8605	BS00051826_51	[**A**/G]	56,751,122	108.87	0.08	0.12	−13.79	7.03E-05	4.15
		QSng.SP20.daw-3A	3A	IWB14389	CAP7_rep_c12940_130	[**T**/C]	646,272,690	346.53	0.07	0.07	−15.26	4.74E-05	4.32
		QSng.SP20.daw-7B	7B	IWB30294	Excalibur_rep_c107796_229	[**T**/C]	105,559,208	229.43	0.09	0.15	−12.80	1.94E-05	4.71
Manjimup 2018 †	Foliar	QSnl.MJ18.daw-1B	1B	IWB49491	Kukri_rep_c111213_148	[**A**/G]	300,949,280	206.69	0.08	0.13	−18.24	5.44E-05	4.26
			1B	IWB72968	Tdurum_contig63991_404	[**T**/C]	301,257,710	206.01	0.10	0.14	−19.32	5.17E-06	5.29
			1B	IWB40986	Kukri_c13156_129	[T/**C**]	301,257,922	206.01	0.08	0.14	17.51	4.57E-05	4.34
			1B	IWB55131	RAC875_c21131_3615	[T/**C**]	302,206,634	206.01	0.08	0.15	17.80	2.72E-05	4.56
			1B	IWB23446	Excalibur_c20228_135	[**T**/C]	305,270,049	206.01	0.09	0.14	−19.09	1.43E-05	4.84
			1B	IWB71062	Tdurum_contig42289_1857	[**A**/C]	306,072,514	206.69	0.08	0.14	−18.34	2.32E-05	4.63
			1B	IWB74187	tplb0024i16_800	[**A**/G]	307,427,828	206.69	0.08	0.14	−17.51	2.92E-05	4.53
			1B	IWB72756	Tdurum_contig60809_268	[T/**G**]	308,587,768	206.01	0.08	0.14	17.08	4.61E-05	4.34
			1B	IWB72755	Tdurum_contig60809_255	[**T**/C]	308,587,781	206.01	0.08	0.14	−17.95	2.97E-05	4.53
			1B	IWB37294	JD_c2834_381	[**T**/C]	309,387,695	206.69	0.09	0.13	−18.57	1.38E-05	4.86
			1B	IWB71413	Tdurum_contig43346_108	[T/**C**]	309,491,071	209.95	0.08	0.13	17.33	5.91E-05	4.23
			1B	IWB63613	RFL_Contig1354_484	[**A**/G]	315,383,705	208.49	0.08	0.17	−15.85	4.37E-05	4.36
			1B	IWB64056	RFL_Contig2784_641	[**A**/G]	317,320,498	206.69	0.08	0.18	−15.57	7.32E-05	4.14
		QSnl.MJ18.daw-5A	5A	IWB35961	IACX448	[**T**/C]	588,377,301	453.34	0.08	0.39	−12.57	6.90E-05	4.16
		QSnl.MJ18.daw-5B	5B	IWB43679	Kukri_c29267_215	[T/**C**]	539,460,125	252.96	0.09	0.07	18.24	1.52E-05	4.82
Manjimup 2019	Foliar	QSnl.MJ19.daw-1A	1A	IWB6426	BS00011521_51	[T/**C**]	579,830,542	431.50	0.07	0.49	9.83	7.65E-05	4.12
		QSnl.MJ19.daw-2B	2B	IWB9450	BS00065105_51	[**T**/C]	69,648,943	262.16	0.07	0.08	−19.36	6.06E-05	4.22
		QSnl.MJ19.daw-4B.1	4B	IWB57527	RAC875_c39524_181	[**A**/G]	126,323,033	182.55	0.10	0.06	−21.86	8.18E-06	5.09
		QSnl.MJ19.daw-4B.2	4B	IWB41569	Kukri_c16392_1468	[**T**/C]	558,051,887	209.83	0.08	0.12	−20.16	5.66E-05	4.25
			4B	IWB38540	Ku_c16392_2687	[A/**C**]	558,053,253	209.83	0.08	0.11	19.30	6.33E-05	4.20
			4B	IWB52053	Ra_c41921_1056	[**T**/G]	558,053,925	209.83	0.09	0.11	−20.63	2.40E-05	4.62
			4B	IWB35570	IAAV8975	[**T**/C]	558,057,833	209.83	0.09	0.12	−22.16	7.61E-06	5.12
			4B	IWB63337	RAC875_rep_c95493_490	[**T**/C]	558,059,510	209.83	0.08	0.10	−20.31	2.58E-05	4.59
			4B	IWB53588	RAC875_c12762_791	[T/**C**]	558,580,422	209.83	0.08	0.07	20.59	4.46E-05	4.35
		QSnl.MJ19.daw-5A	5A	IWB7820	BS00031117_51	[**T**/C]	588,375,856	453.34	0.07	0.42	−13.46	7.21E-05	4.14
Manjimup 2020	Foliar	QSnl.MJ20.daw-1A.1	1A	IWB45604	Kukri_c46010_872	[T/**G**]	32,894,182	ND	0.08	0.12	11.12	1.30E-05	4.89
			1A	IWB26996	Excalibur_c4887_1814	[T/**C**]	35,533,570	184.34	0.07	0.16	9.45	5.64E-05	4.25
		QSnl.MJ20.daw-1A.2	1A	IWB10491	BS00070695_51	[T/**C**]	586,914,453	462.61	0.11	0.45	8.95	7.30E-07	6.14
		QSnl.MJ20.daw-5B	5B	IWA7227	wsnp_Ku_c6464_11320381	[T/**C**]	402,843,711	152.47	0.09	0.34	8.53	1.58E-05	4.80
			5B	IWB8558	BS00049793_51	[T/**G**]	402,843,834	152.47	0.08	0.37	8.39	5.01E-05	4.30
South Perth 2020	Foliar	QSnl.SP20.daw-3D	3D	IWB17658	D_F5XZDLF02HWOJZ_227	[**A**/G]	31,764,661	284.57	0.07	0.05	−24.50	6.69E-05	4.17
		QSnl.SP20.daw-6D	6D	IWB70297	Tdurum_contig31718_229	[T/**C**]	307,881,449	183.08	0.08	0.36	12.53	2.35E-05	4.63
			6D	IWB36455	Jagger_c1746_113	[**T**/C]	307,882,817	183.08	0.08	0.36	−12.00	5.53E-05	4.26
		QSnl.SP20.daw-7A	7A	IWB52779	Ra_c8937_191	[A/**G**]	81,498,302	332.69	0.08	0.27	13.66	5.47E-05	4.26
			7A	IWB64358	RFL_Contig3447_1177	[A/**G**]	81,498,578	332.69	0.07	0.27	13.46	6.22E-05	4.21

*All SNP markers within QTL have moderate to high level of significance based on threshold values of *P*<7.65 × 10^–5^ (−log_10_(*p*) > 4.12). ^*a*^Desirable SNP for reduced disease, based on the allele effect estimate, is in bold and underlined. ^*b*^Base pair (bp) location of the single base change in the IWGSC RefSeq v1.0. ^*c*^Consensus map position as reported in Wang et al. (2014), cM: centimorgans. ^*d*^MAF: minor allele frequency. ^*e*^The effect estimates the difference between the average phenotypic values of the homozygous A genotype relative to the homozygous B genotype. ^†^Data previously reported in Francki et al. (2020). ND, Not determined.*

At total of 14 QTL were detected for foliar response in trials at Manjimup and South Perth in 2018–2020 (Table 7). There were SNP markers 1445 bp apart that detected QTL at Manjimup in 2018 and 2019, *QSnl.MJ18.daw-5A* and *QSnl.MJ19.daw-5A* (Table 7), indicating QTL are co-located on chromosome 5A. The remaining QTL for foliar response were detected in only one environment and, therefore, were determined as environment-specific (Table 7). The estimated allelic effects ranged from 8.39 to 24.50% (Table 7). The physical position of SNP markers associated with heading date and plant height (Supplementary Table 5) were not co-located or in LD and, therefore, were not considered to be associated with foliar response.

**FIGURE 1:**
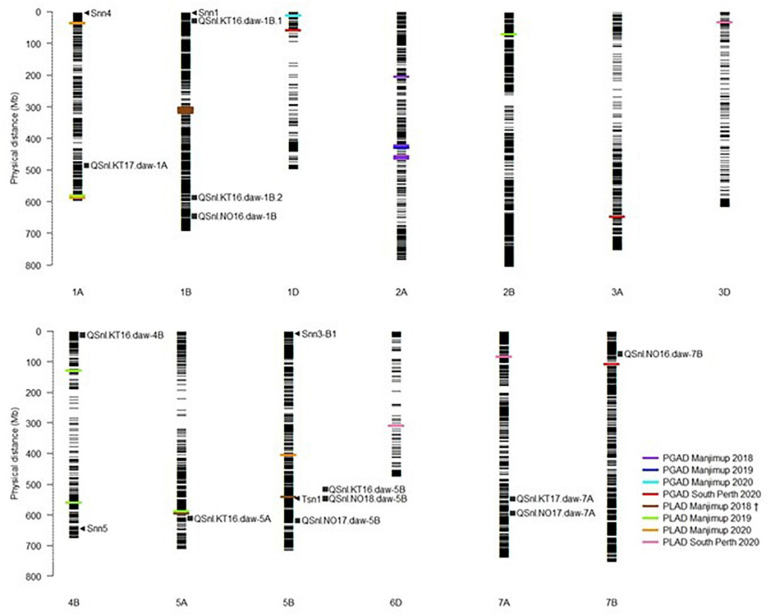
Comparison of QTL for PGAD and PLAD resistance. Assignment of known *Tsn* and *Sn*n loci and position of MTA detected in multiple environments in 2018–2020 on the Chinese Spring physical map (IGWSC RefSeq v1.0). Black horizontal lines represent the physical locations (Mb) of SNP markers used in GWAS analysis. Colored bars represent the MTA detected in different environments in 2018–2020. Arrows indicate putative location of known *Snn* and *Tsn1* loci. Squares indicate QTL for foliar SNB resistance detected in 2016–2018 and reported in Francki et al., 2020. ^†^PLAD Manjimup 2018 also reported in Francki et al. (2020).

To ascertain a genetic relationship with glume response, it was necessary to detect genomic regions for foliar response and compare the position of markers associated with respective QTL on the wheat physical map. A genetic relationship between glume and foliar response was recognized if QTL for each trait were either co-located or in LD. A comparison based on the physical map position of associated SNP markers indicated that QTL for glume and foliar response neither co-located nor were in LD within or between environments in 2018–2020 (Table 7 and Figure 1). Furthermore, QTL detected in this study were not in LD with other QTL for foliar response detected in other WA environments (Francki et al., 2020; Figure 1). The lack of QTL co-located or in LD makes it reasonable to assume, therefore, that glume and foliar responses to SNB are controlled by multiple but independent genes that respond in specific environments.

*Snn* loci were positioned on physical chromosome maps with QTL for glume and foliar response detected in 2018–2020. *Snn4*, *Snn1*, *Snn5* were mapped on chromosomes 1A, 1B, and 4B, respectively while both *Snn3-B1* and *Tsn1* mapped to chromosome 5B (Figure 1). Neither QTL for glume nor foliar response detected across four environments in 2018–2020 were in LD to the *Snn* loci based on physical map position, indicating that interactions with known NE were not evident in any field environments in 2018–2020. The exception was *QSnl.MJ18.daw-5B* in LD with *Tsn1* (Figure 1) previously reported in Francki et al. (2020).

## Discussion

There is increasing evidence that disease response to glume and foliar SNB in the field is controlled by many independent and mostly environmental-specific QTL (Ruud and Lillemo, 2018; Czembor et al., 2019; Ruud et al., 2019; Francki et al., 2020; Lin et al., 2020) exacerbating the complexity of genetic resistance and susceptibility to SNB in wheat. The majority of the QTL detected for either glume or foliar response to SNB in this study were detected at one location but not another, confirming the inherent and convoluted genetic mechanisms for resistance and susceptibility in field assessment. The outcome of this study confirms an independent genetic relationship between glume and foliar response when wheat lines were evaluated at any particular location, evident by the lack of SNP markers associated with QTL that were neither co-located nor in LD. It is assumed, therefore, corresponding genes for biological mechanisms underpinning resistance and susceptibility to pathogen infection and disease progression are dissimilar in glumes and foliage whereby several host genes may be influenced by developmental stages and host-isolate-environmental interactions.

It was presumed that environment-isolate interactions could have a significant effect on host genes responding to SNB in WA (Francki et al., 2020). This study monitored climatic conditions in successive years at Manjimup in 2018–2020 and showed similar daily average air temperature, relative humidity, rainfall, solar exposure and pan evaporation. On the contrary, South Perth in 2020 had higher daily average air temperature, solar exposure and pan evaporation but lower rainfall and relative humidity than any of the Manjimup environments. Therefore, it was expected that SNB response across 232 wheat lines would be consistent across Manjimup environments but variable to South Perth. Although climate impacted disease progression between Manjimup and South Perth sites, there was insubstantial effects for disease response in 2018–2020 evident through high phenotypic correlations and low environment interactions. However, this conclusion is in contrast to moderate correlation reported for foliar response of wheat genotypes across six WA environments in 2016–2018 (Francki et al., 2020). Differences in aggressiveness due to isolate-by-environment interactions (Pariaud et al., 2009; Sharma and Verma, 2019) could partly explain variable SNB response across environments in 2016–2018 (Francki et al., 2020). Since isolates in this study were different to those reported in Francki et al. (2020) it is plausible, therefore, aggressiveness of isolates selected for this study could be less affected by environmental variables in 2018–2020. Alternatively, several but different host loci from diverse germplasm may respond independently to varying levels of aggressiveness of isolates (Krupinksy, 1997) and may account for higher phenotypic correlations between environments. There is a need, therefore, for increased knowledge on the significance of specific environment-by-isolate and genotype-by-isolate interactions and their effects on host quantitative genetic resistance to provide a holistic perception of the tripartite interaction central for glume and foliar SNB disease response in different field environments.

Finlay-Wilkinson regression can reveal trends of variety performance across environments and select lines either based on stability or on responsiveness to environment potential (Finlay and Wilkinson, 1963). Evaluation of 232 wheat lines for glume response to SNB across four environments identified 35 lines that showed PGAD scores <20% and are resistance donors for breeding glume blotch resistance. EGA Bonnie Rock and ZWW09Qno177 were of particular interest because of their high stability and predictability for glume resistance across multiple field environments, where the former also showed consistently low mean PLAD scores and stability across multiple WA environments. Included in the panel were eight lines with low foliar response when evaluated against 42 different isolates across six environments in 2016–2018 (Francki et al., 2020) which indicated sustained foliar resistance but with varying degrees of stability. The phenotypic correlation between glume and foliar response in the GWAS population was generally higher within each environment to those previously reported for bi- or multi-parental populations evaluated in Australia (Shankar et al., 2008), Europe (Wicki et al., 1999; Aguilar et al., 2005) and Nordic regions (Lin et al., 2020).

We further explored the genetic relationship between glume and foliar response in any particular environment by projecting SNP markers associated with QTL on the physical map and identifying those co-located or in LD to assess if there was common genetic control for these traits. Despite eight QTL for glume and 14 for foliar resistance detected, none were either co-located or in LD within or between four environments. Therefore, GWAS using higher resolution genetic mapping confirmed that genetic control for glume and foliar response is independent even though high phenotypic correlation was observed across environments for each trait. High correlation for glume and foliar response may be attributed to the cumulative influence of different QTL having phenotypic effects in specific environments, including any with small effects that may not have been detected in this study due to the lack of statistical power in GWAS analysis. Interestingly, the cumulative influence of environment-specific QTL of small and larger effect contributing to high phenotypic correlation across Australian environments was recently reported for durable rust resistance (Joukhadar et al., 2020). The increased number of loci detected for both traits and better precision in mapping of alleles using GWAS gives particular credence to the independent control of glume and foliar response and the potential role of cumulative but small effect of environment-specific QTL in SNB response. Independent loci with small phenotypic effects controlling glume and foliar response is in agreement with previous studies evaluating bi-parental and multi-parental mapping populations (Fried and Meister, 1987; Bostwick et al., 1993; Wicki et al., 1999; Shankar et al., 2008; Czembor et al., 2019; Lin et al., 2020).

QTL for heading date and height were not co-located or in LD with any QTL for glume response. Therefore, it is reasonable to assume that the QTL represented true resistance rather than a pleiotropic effect of heading date and plant height. The majority of QTL for glume resistance in this study were detected in one environment only. The exception was a QTL on chromosome 2A at 423.20 Mbp detected at Manjimup in 2018 and 2019 (*QSng.MJ18.daw-2A.2* and *QSng.MJ19.daw-2A*, respectively) with a large allele effect estimate of 20.63% for average phenotypic values indicating the same QTL may be effective in different but not all environments. Interestingly, the nature of QTL for glume resistance in this study was in agreement with previous reports in that some were detected in only one environment (Shankar et al., 2008; Czembor et al., 2019; Lin et al., 2020) while only a few QTL in the same genomic region were detected across multiple environments (Schnurbusch et al., 2003; Uphaus et al., 2007; Shankar et al., 2008; Lin et al., 2020). QTL for glume resistance has not been previously identified on chromosome 1D, so it appears that *QSng.MJ20.daw-1D* and *QSng.SP20.daw-1D* are novel and accentuates the importance of evaluating wider germplasm pools to identify new sources of variation suitable for breeding glume blotch resistance. A comparison of the physical position of SNP markers associated with QTL for glume response on chromosome 2A, 3A and 7B were neither co-located nor in LD with QTL for glume resistance reported by Lin et al. (2020) confirming the minor and different gene effects for SNB response in earlier studies (Fried and Meister, 1987; Wicki et al., 1999. The physical co-location of QTL for glume resistance previously reported on chromosomes 2A (Schnurbusch et al., 2003), 3A (Schnurbusch et al., 2003; Aguilar et al., 2005) and 7B (Schnurbusch et al., 2003) was not readily discernible due to ambiguous positioning of markers and, consequently, validation for the same genomic regions controlling glume response between populations was inconclusive.

Similar to glume response, QTL for morphological traits did not co-locate or were in LD with QTL for foliar response so it appears that loci detected are specific to SNB disease. We used SNP markers associated with foliar resistance to SNB in adult plants from other studies, wherever possible, to anchor and validate QTL and compare their location on the physical map. Foliar QTL detected in 2018–2020 other than *QSnl.MJ18.daw-1B* neither co-located nor were in LD with previous QTL detected when the population was evaluated in WA environments (Francki et al., 2018, 2020). However, some QTL including *QSnl.MJ18.daw-1A, QSnl*.MJ20.daw-1A.2 and *QSnl.MJ19.daw-2B* were either co-located or in LD with similar genomic regions controlling foliar resistance on chromosomes 1A, and 2B reported by Ruud et al. (2019). It is reasonable to assume, therefore, that these QTL are within common genomic regions that harbor genes controlling SNB response in different regions of the world and presumably genetically different isolates. Similar to the comparison for glume resistance, it was not discernible to accurately validate existing or identify novel QTL for some foliar SNB resistance on the physical map from earlier studies (Schnurbusch et al., 2003; Aguilar et al., 2005; Friesen et al., 2009; Czembor et al., 2019) mainly due to low resolution genetic mapping and ambiguous anchoring of markers other than SNPs. Nevertheless, a myriad of loci responded to foliar SNB infection in an environmental-specific manner and/or as a result of variability in pathogen isolates.

High abundance of SNP markers discriminated co-located QTL from low resolution genetic mapping into separate but closely accompanying QTL may contain clusters of concomitant disease-related genes for glume and foliar SNB resistance (Francki et al., 2018) with increasing evidence from recent GWAS studies that these clusters respond to pathogen infection in a genotype-by-environment-by isolate manner (Francki et al., 2020). This study identified accompanying QTL for glume resistance separated by a physical distance of ∼30 Mbp on chromosome 2A, *QSng.MJ18.daw-2A.2* and *QSng.MJ18.daw-2A.3*, providing further evidence that some genes responding to SNB are within distinct clusters on chromosomes. Likewise, a pair of QTL for foliar response in LD were detected on chromosome 1A in regions 579.83 Mbp to 586.91 Mbp (*QSnl.MJ19.daw-1A* and *QSnl.MJ20.daw-1A.2*, respectively) and within a 1,445 bp region on 5A around 588.37 Mbp (*QSnl.MJ18.daw-5A* and *QSnl.MJ19.daw-5A*) providing further credibility that clusters of genes reside within a small physical distance and respond to different environments and/or isolates. Sequence analysis will reveal whether the region on 1D and 1A contain related disease resistance gene classes and whether the QTL on 5A has one or tandem genes.

It was expected that QTL detected using GWAS would identify those co-located or in LD with known *Snn* and *Tsn1* loci particularly in genomic regions with high-density SNP markers for loci on chromosomes 1A, 1B, 4B and 5B. Although the physical location of *Snn4*, *Snn1*, *Snn5* were located on chromosomes 1A, 1B and 4B respectively and *Snn3-B1* and *Tsn1* mapping to chromosome 5B, the QTL for glume and foliar response were not in LD with *Snn* loci. The only exception was *QSnl.MJ18.daw-5B* previously identified to be in LD with *Tsn1* on 5B (Francki et al., 2020). Therefore, it does not appear that known NE-*Snn* interactions have a prominent effect on glume or foliar disease when wheat was evaluated in any of the four WA environments in 2018–2020. This may be due to different NE genes in isolates, variations in *Snn* and *Tsn* sensitivity genes represented in the 232 wheat lines and/or different environmental effects that influence compatible NE-*Snn* interactions for disease progression in the field. Nevertheless, taken collectively with multiple field evaluation in Francki et al. (2020), this study validated that known NE-*Snn* interactions do not appear to influence quantitative glume and foliar resistance in WA environments, a supposition shared in other studies when wheat was evaluated for SNB response in the eastern region of the United States (Cowger et al., 2020) and Europe (Czembor et al., 2019). We cannot exclude the possibility that undetected NE-*Snn* interactions may serve a role in SNB response in wheat. If so, a myriad of interactions would be assumed given that multiple and environment-specific loci contribute to glume and foliar response. The importance of increasing our knowledge on the genetic diversity of isolates, the interaction of environmental effects on pathogenicity and aggressiveness and on host genes would play a critical role in deciphering the biological mechanisms underpinning glume and foliar response to SNB. In the meantime, breeding for improved SNB resistance in wheat remains a challenging task. Enrichment of resistance alleles using SNP markers identified in this study may contribute minor effects in specific environments but development of breeding lines with robust resistance would significantly benefit by recurrent phenotypic selection against different isolates and multiple environments. Developing a genomic selection breeding strategy would be a worthwhile proposition but would require multi-environment trial, biological and biophysical environmental information for modeling and deriving accurate prediction equations.

## Conclusion

The majority of QTL for glume resistance to SNB were environmentally-specific in four environments and provided further understanding of genotype-by-environment interactions. Moreover, QTL for glume resistance did not coincide with foliar resistance confirming the added complexity of different genotype-pathogen-environment interactions and underpinning biological pathways leading to alternative SNB responses in adult plants. GWAS did not detect QTL co-located or in LD with known *Snn* or *Tsn* loci so their role in controlling either glume or foliar response to SNB is not apparent in the environments selected in this study. It is important, however, to consider further research on potentially different disease response pathways to gain a better understanding on fundamental biology underpinning resistance and susceptibility. In the meantime, strategies for breeding will rely on recurrent phenotypic evaluation to capture and retain favorable alleles for both glume and foliar resistance relevant to the particular environment.

## Data Availability Statement

Requests to access the datasets should be directed to the corresponding author.

## Author Contributions

MF acquired research funding, designed experiments, collated, analyzed and interpreted data, and wrote the manuscript. EW contributed to data acquisition, analysis and interpretation, and contributed to writing the manuscript. CM and WM contributed to trial designs, planting, maintenance, and data acquisition. All authors read and approved the final manuscript.

## Conflict of Interest

The authors declare that the research was conducted in the absence of any commercial or financial relationships that could be construed as a potential conflict of interest.

## Footnotes

1 https://weather.agric.wa.gov.au/

2 https://genstat.kb.vsni.co.uk

3 https://urgi.versailles.inra.fr/
