# Supplementary material for: Evaluation of Septoria Nodorum Blotch (SNB) Resistance in Glumes of Wheat (Triticum aestivum L.) and the Genetic Relationship With Foliar Disease Response
Source: Front Genet. 2021 Jun 29;12:681768. doi: 10.3389/fgene.2021.681768 (PMC8276050; doi:10.3389/fgene.2021.681768)
Supplement: Supplementary file 1 [file Presentation_1.ZIP › Figure S1.PDF]

SUPPLEMENTARY FIGURE 1| Frequency distribution of percent glume area disease (PGAD) and percent leaf area disease (PLAD) for 232 hexaploid wheat accessions evaluated at Manjimup (MJ) and South Perth (SP) in 2018-2020.

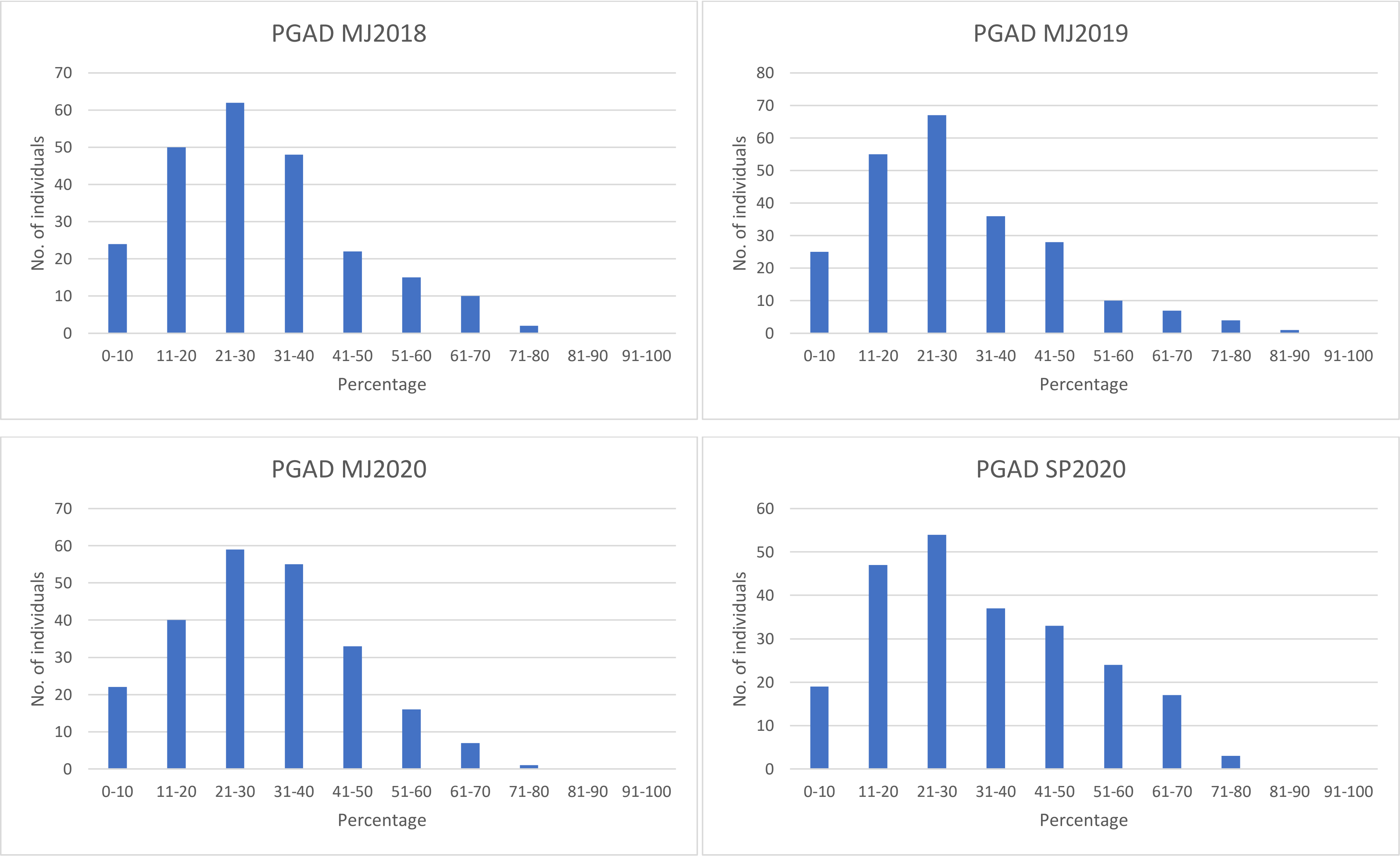

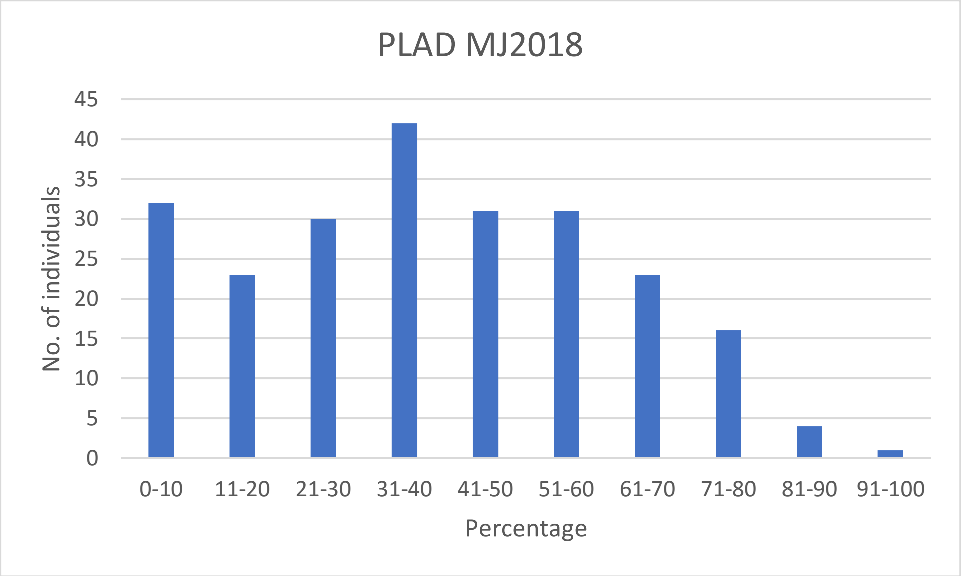
PLAD MJ2018
45
40
35
30
25
20
15
10
5
0
No. of individuals
0-10
11-20
21-30
31-40
41-50
51-60
61-70
71-80
81-90
91-100
Percentage

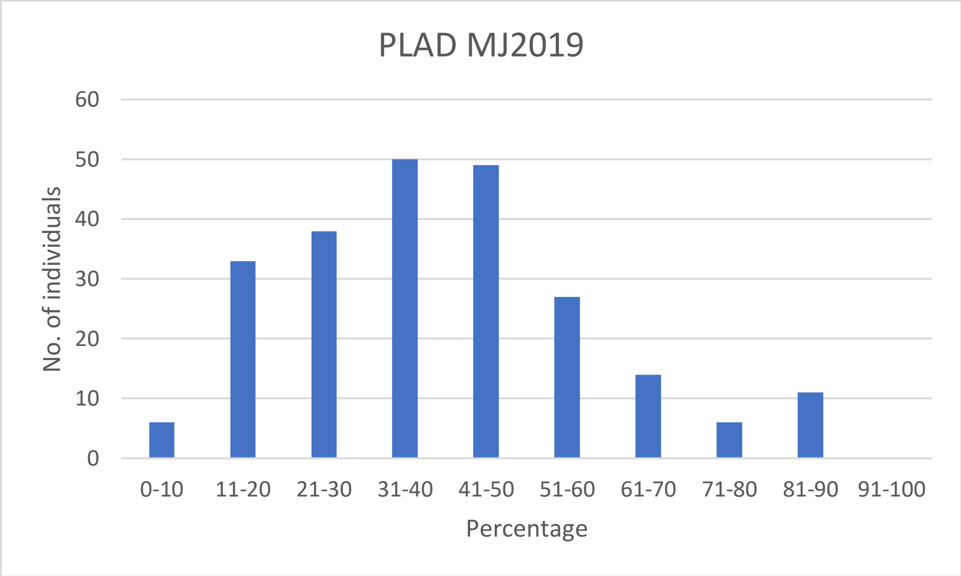
PLAD MJ2019
60
50
40
30
20
10
0
No. of individuals
0-10
11-20
21-30
31-40
41-50
51-60
61-70
71-80
81-90
91-100
Percentage

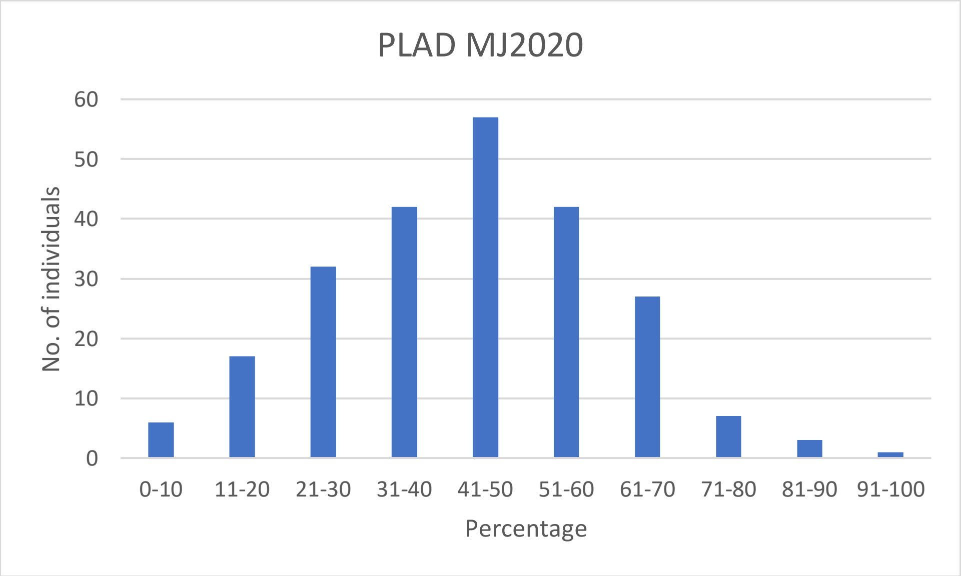
PLAD MJ2020
60
50
40
30
20
10
0
No. of individuals
0-10
11-20
21-30
31-40
41-50
51-60
61-70
71-80
81-90
91-100
Percentage

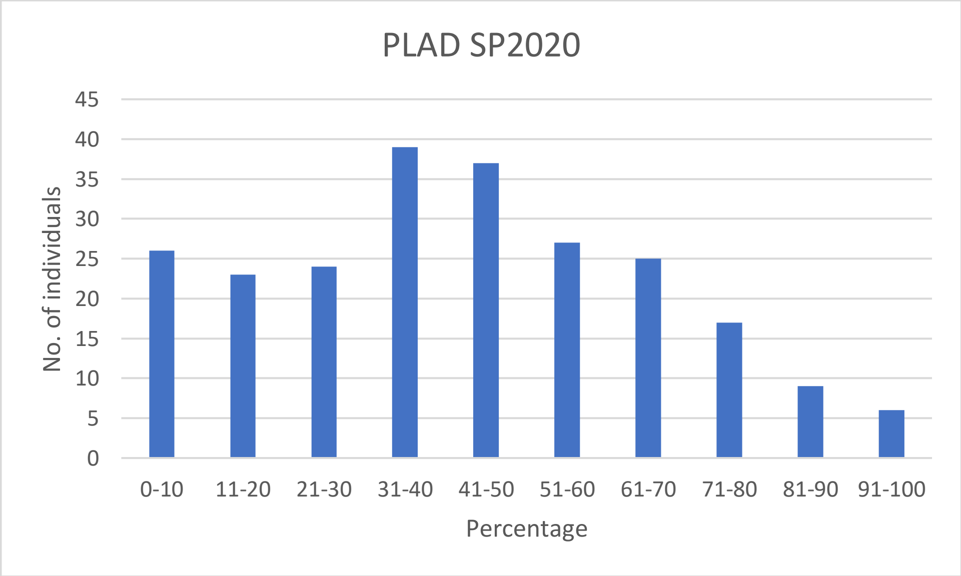
PLAD SP2020
45
40
35
30
25
20
15
10
5
0
No. of individuals
0-10
11-20
21-30
31-40
41-50
51-60
61-70
71-80
81-90
91-100
Percentage
