# Supplementary figures and images for: Evaluation of Septoria Nodorum Blotch (SNB) Resistance in Glumes of Wheat (Triticum aestivum L.) and the Genetic Relationship With Foliar Disease Response
Source: Front Genet. 2021 Jun 29;12:681768. doi: 10.3389/fgene.2021.681768 (PMC8276050; doi:10.3389/fgene.2021.681768)

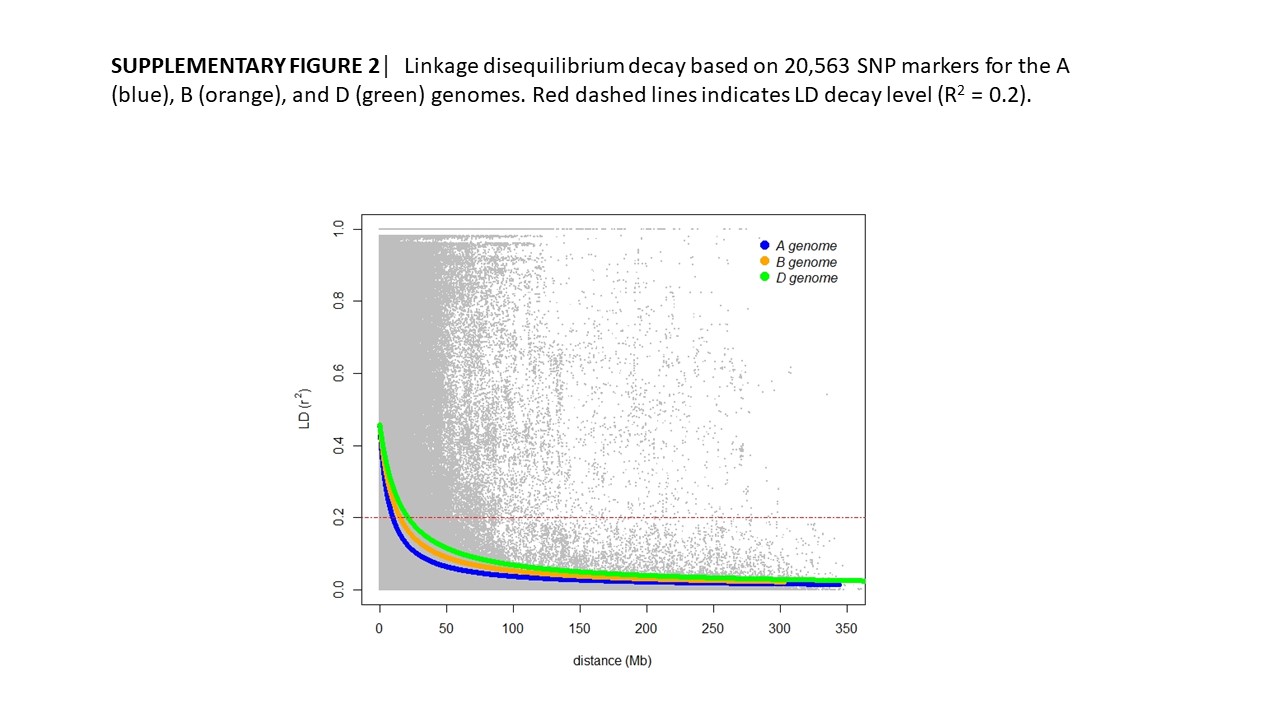

Supplement: Supplementary file 1 [file Presentation_1.ZIP › Figure S2.JPEG]

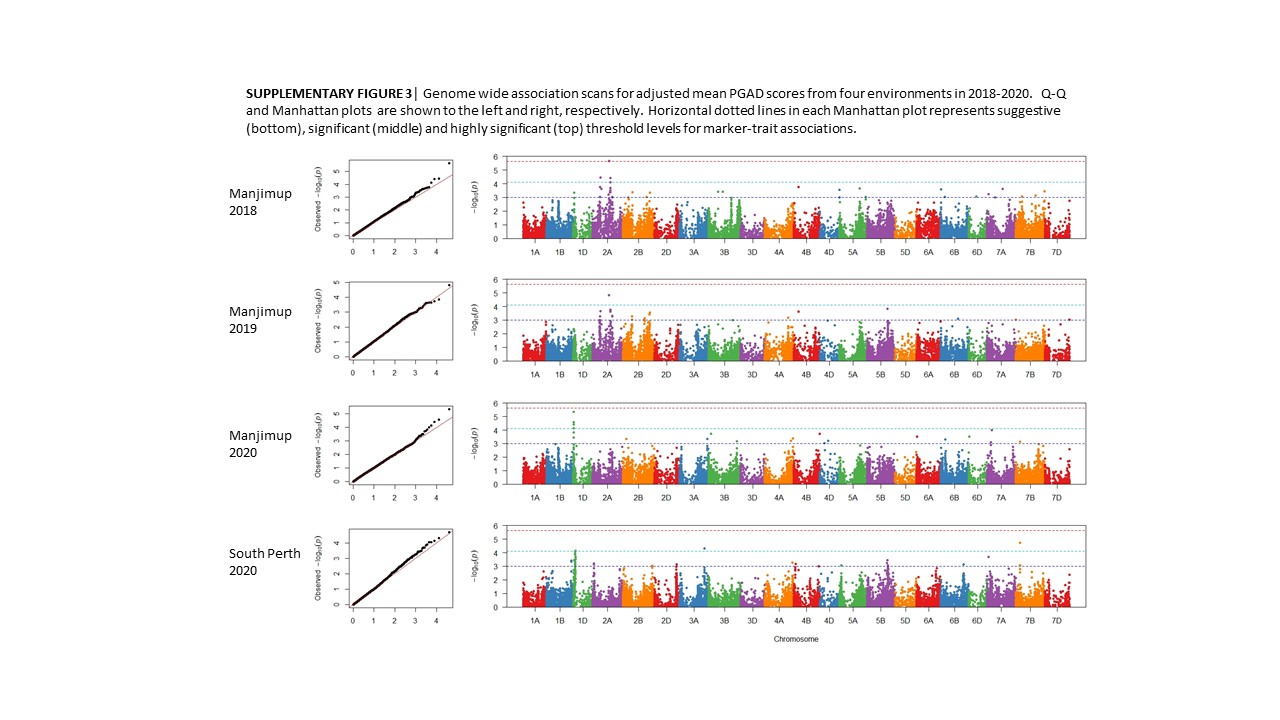

Supplement: Supplementary file 1 [file Presentation_1.ZIP › Figure S3.JPEG]

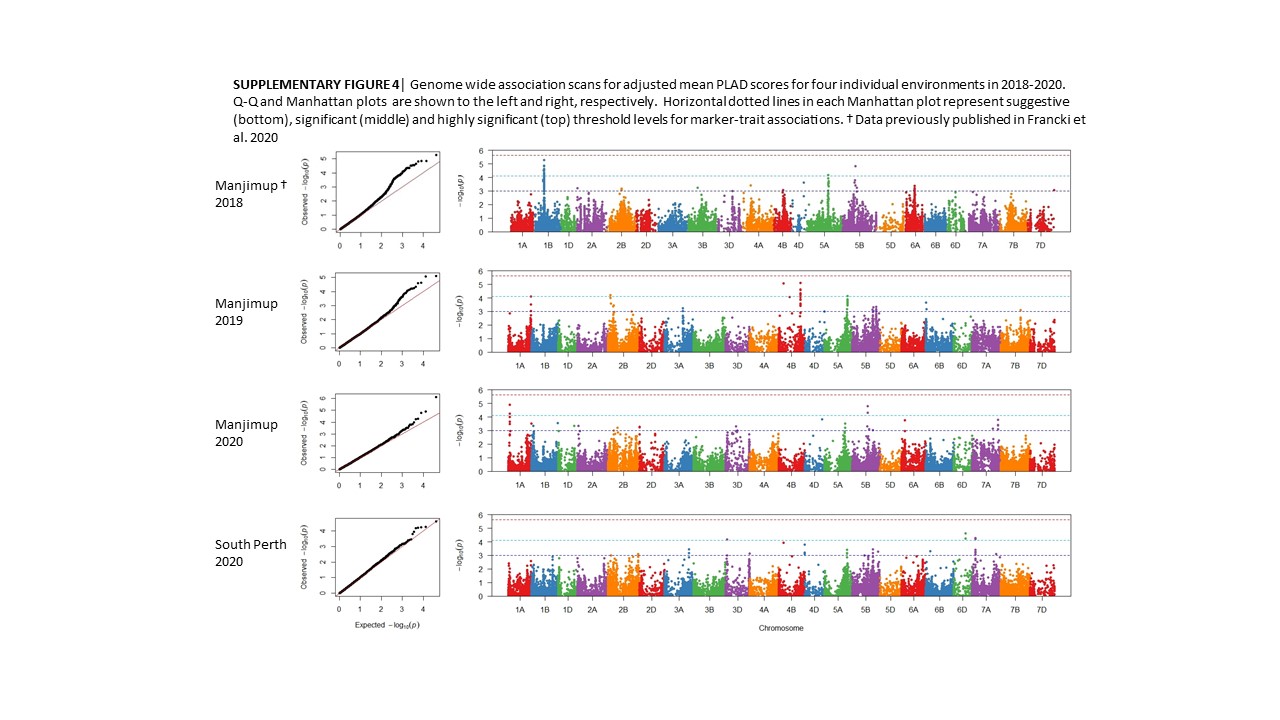

Supplement: Supplementary file 1 [file Presentation_1.ZIP › Figure S4.JPEG]
