# Supplementary material for: Evaluation of Septoria Nodorum Blotch (SNB) Resistance in Glumes of Wheat (Triticum aestivum L.) and the Genetic Relationship With Foliar Disease Response
Source: Front Genet. 2021 Jun 29;12:681768. doi: 10.3389/fgene.2021.681768 (PMC8276050; doi:10.3389/fgene.2021.681768)
Supplement: Supplementary file 3 [file Table_2.DOCX]

| **2018 trial** | | | **2019 trials** | | | **2020 trials** | | |
| --- | --- | --- | --- | --- | --- | --- | --- | --- |
| Isolate | Year | Location | Isolate | Year | Location | Isolate | Year | Location |
| **WAC13077** | 2005 | Geraldton | WAC8635 | 1994 | Unknown | WAC8635 | 1994 | Unknown |
| **WAC13206** | 2008 | South Perth | **WAC13077** | 2005 | Geraldton | **WAC13077** | 2005 | Geraldton |
| WAC13524 | 2011 | South Perth | **WAC13206** | 2008 | South Perth | **WAC13206** | 2008 | South Perth |
| WAC 13690 | 2014 | Dangara | WAC 13404 | 2011 | South Perth | WAC 13404 | 2011 | South Perth |
| WAC 13741 | 2014 | Katanning | WAC13524 | 2011 | South Perth | WAC 13691 | 2014 | Dongara |
| **WAC13872** | 2015 | Geraldton | WAC13667 | 2016 | Merredin | **WAC13872** | 2015 | Geraldton |
| WAC13957 | 2016 | South Perth | WAC 13690 | 2014 | Dongara | WAC13959 | 2016 | South Perth |
| WAC13967 | 2016 | Merredin | WAC 13691 | 2014 | Dongara | WAC14138 | 2018 | Geraldton |
| WAC13969 | 2016 | Wongan Hills | **WAC13872** | 2015 | Geraldton | WAC 14322 | 2019 | South Perth |
| WAC13979 | 2017 | Wongan Hills | WAC13959 | 2016 | South Perth | WAC 14323 | 2019 | South Perth |
| WAC14056 | 2017 | Wongan Hills | WAC14057 | 2017 | Wongan Hills | WAC 14324 | 2019 | Manjimup |
| WAC14057 | 2017 | Wongan Hills | WAC14061 | 2017 | Geraldton | WAC 14325 | 2019 | Manjimup |
| WAC14058 | 2017 | Geraldton | WAC14138 | 2018 | Geraldton |  |  |  |
| WAC14059 | 2017 | Geraldton | WAC14140 | 2018 | Narrogin |  |  |  |
| WAC14060 | 2017 | Geraldton | WAC14141 | 2018 | Wongan Hills |  |  |  |
| WAC14061 | 2017 | Geraldton | WAC14142 | 2018 | Wongan Hills |  |  |  |
| WAC14062 | 2017 | Wongan Hills | WAC14143 | 2018 | Corrigin |  |  |  |
| WAC14066 | 2017 | Northam |  |  |  |  |  |  |
| WAC14067 | 2017 | Northam |  |  |  |  |  |  |
|  |  |  |  |  |  |  |  |  |

**SUPPLEMENTARY TABLE 2│** *Parastagonospora nodorum* isolate description used for field evaluation of wheat genotypes in 2018-2020. Year and geographical location of isolates collected in Western Australia is provided. Common isolates used in each year are highlighted in bold.
