# Supplementary material for: Evaluation of Septoria Nodorum Blotch (SNB) Resistance in Glumes of Wheat (Triticum aestivum L.) and the Genetic Relationship With Foliar Disease Response
Source: Front Genet. 2021 Jun 29;12:681768. doi: 10.3389/fgene.2021.681768 (PMC8276050; doi:10.3389/fgene.2021.681768)
Supplement: Supplementary file 4 [file Table_3.DOCX]

|  |  | **Min Temp^a^** | **Max Temp^a^** | **Min RH^b^** | **Max RH^b^** | **Rainfall** | **Solar exposure** | **Pan evaporation** |
| --- | --- | --- | --- | --- | --- | --- | --- | --- |
| **Manjimup 2018** | Min | 2.8^o^C | 9.7 ^o^C | 27.6% | 78.1% | 0.0 mm | 3.6 MJ | 0.5 mm |
|  | Max | 12.8 ^o^C | 24.4 ^o^C | 90.0% | 99.7% | 31.2 mm | 22.9 MJ | 5.4 mm |
|  | Daily average | 8.1 ^o^C | 15.5 ^o^C | 61.3% | 95.4% | 4.8 mm | 11.1 MJ | 1.9 mm |
| **Manjimup 2019** | Min | 2.5 ^o^C | 11.6 ^o^C | 20.7% | 67.3% | 0.0 mm | 1.7 MJ | 0.0 mm |
|  | Max | 14.8 ^o^C | 27.1 ^o^C | 88.4% | 99.7% | 45.0 mm | 21.6 MJ | 5.5 mm |
|  | Daily average | 8.0 ^o^C | 16.3 ^o^C | 61.1% | 94.8% | 4.2 mm | 11.1 MJ | 2.0 mm |
| **Manjimup 2020** | Min | 2.7 ^o^C | 11.0 ^o^C | 40.3% | 72.7% | 0.0 mm | 2.9 MJ | 0.2 mm |
|  | Max | 14.1 ^o^C | 21.7 ^o^C | 88.3% | 99.6% | 46.8 mm | 24.2 MJ | 4.8 mm |
|  | Daily average | 7.9 ^o^C | 16.1 ^o^C | 59.3% | 95.7% | 4.2 mm | 12.0 MJ | 2.2 mm |
| **South Perth 2020** | Min | 4.3 ^o^C | 12.2 ^o^C | 15.0% | 55.6% | 0.0 mm | 3.9 MJ | 0.4 mm |
|  | Max | 17.0 ^o^C | 29.4 ^o^C | 77.1% | 97.9% | 32.6 mm | 26.8 MJ | 7.6 mm |
|  | Daily average | 10.0 ^o^C | 20.0 ^o^C | 45.1% | 90.9% | 2.9 mm | 13.8 MJ | 3.0 mm |

**SUPPLEMENTARY TABLE 3│** Climate measurements at four locations during disease progression from first inoculation at Feekes 3-5 to recording of glume scores at ripening (Feekes 10.1).

^a^Air temperature

^b^Relative humidity
