# Supplementary material for: Evaluation of Septoria Nodorum Blotch (SNB) Resistance in Glumes of Wheat (Triticum aestivum L.) and the Genetic Relationship With Foliar Disease Response
Source: Front Genet. 2021 Jun 29;12:681768. doi: 10.3389/fgene.2021.681768 (PMC8276050; doi:10.3389/fgene.2021.681768)
Supplement: Supplementary file 5 [file Table_4.DOCX]

|  | **Thermal time** | | |
| --- | --- | --- | --- |
|  |  | Glume score (^o^Cd) | Foliar score (^o^Cd) |
| Manjimup 2018 |  | 1213 | 969 |
| Manjimup 2019 |  | 1117 | 972 |
| Manjimup 2020 |  | 1238 | 1074 |
| South Perth 2020 |  | 1589 | 1499 |

**SUPPLEMENTARY TABLE 4│** Thermal time for SNB progression from first day of inoculation to the day of disease evaluation at four WA environments.
