# Supplementary material for: Evaluation of Septoria Nodorum Blotch (SNB) Resistance in Glumes of Wheat (Triticum aestivum L.) and the Genetic Relationship With Foliar Disease Response
Source: Front Genet. 2021 Jun 29;12:681768. doi: 10.3389/fgene.2021.681768 (PMC8276050; doi:10.3389/fgene.2021.681768)
Supplement: Supplementary file 6 [file Table_5.DOCX]

**SUPPLEMENTARY TABLE 5 │**Summary of SNP marker associations for heading date (HD) and plant height (PH). All SNP markers within QTL have moderate to high level of significance based on threshold values of *p* < 7.65x10^-5^ (-log_10_(*p*) > 4.12).

| **Environment** | **Trait** | **QTL** | **Chromosome** | **SNP id** | **SNP name** | **SNP^a^** | **IWGSC-bp^b^** | **Consensus map position-cM^c^** | **R^2^** | **MAF^d^** | **Allele effect estimate %^e^** | ***p*-value** | **-log_10_(*p*)** |
| --- | --- | --- | --- | --- | --- | --- | --- | --- | --- | --- | --- | --- | --- |
| Manjimup 2018 | HD | *QHd.MJ18.daw-1B* | 1B | IWB72443 | Tdurum_contig55429_116 | [T/C] | 664,302,556 | 428.89 | 0.10 | 0.05 | 12.35 | 6.47E-06 | 5.19 |
| Manjimup 2019 | HD | NA |  |  |  |  |  |  |  |  |  |  |  |
| Manjimup 2020 | HD | *QHd.MJ20.daw-2D* | 2D | IWA6302 | wsnp_JD_rep_c63957_40798121 | [T/C] | 20,768,520 | 32.08 | 0.09 | 0.44 | -4.61 | 1.61E-05 | 4.79 |
|  |  | *QHd.MJ20.daw-5B* | 5B | IWB36002 | IACX5702 | [A/G] | 577,227,941 | 333.02 | 0.09 | 0.22 | -5.63 | 6.23E-05 | 4.21 |
| South Perth 2020 | HD | *QHd.SP20.daw-1B* | 1B | IWB68743 | Tdurum_contig18557_228 | [A/G] | 664,285,795 | 428.89 | 0.08 | 0.05 | 7.79 | 6.14E-05 | 4.21 |
|  |  |  | 1B | IWB72443 | Tdurum_contig55429_116 | [T/C] | 664,302,556 | 428.89 | 0.11 | 0.05 | 9.56 | 3.93E-06 | 5.41 |
|  |  | *QHd.SP20.daw-3D* | 3D | IWA153 | wsnp_BE444579D_Ta_2_3 | [A/G] | 604,366,294 | 416.72 | 0.09 | 0.06 | 7.80 | 3.85E-05 | 4.41 |
| Manjimup 2018 | PH | *QHt.MJ18.daw-4B* | 4B | IWB6961 | BS00022194_51 | [T/C] | 475,806,782 | 193.52 | 0.09 | 0.05 | -10.75 | 1.18E-05 | 4.93 |
|  |  |  | 4B | IWB72211 | Tdurum_contig51818_145 | [A/G] | 475,806,882 | 193.52 | 0.09 | 0.06 | -10.74 | 8.29E-06 | 5.08 |
|  |  |  | 4B | IWB26339 | Excalibur_c42450_727 | [T/C] | 479,154,233 | 195.17 | 0.08 | 0.07 | -9.30 | 6.24E-05 | 4.21 |
| Manjimup 2019 | PH | *QHt.MJ19.daw-1B* | 1B | IWB65905 | TA004946-0577 | [T/C] | 640,560,372 | 356.34 | 0.09 | 0.07 | -8.77 | 2.27E-05 | 4.64 |
|  |  | *QHt.MJ19.daw-2A* | 2A | IWB40126 | Ku_c8927_2075 | [A/G] | 691,217,702 | 413.64 | 0.09 | 0.23 | -6.02 | 7.06E-05 | 4.15 |
|  |  | *QHt.MJ19.daw-3A* | 3A | IWB64831 | RFL_Contig4921_2352 | [T/C] | 564,944,064 | ND | 0.08 | 0.38 | 4.67 | 3.04E-05 | 4.52 |
| Manjimup 2020 | PH | NA |  |  |  |  |  |  |  |  |  |  |  |
| South Perth 2020 | PH | *QHt.SP20.daw-5D* | 5D | IWB54648 | RAC875_c18322_670 | [A/G] | 31,273,513 | ND | 0.09 | 0.06 | -7.46 | 9.66E-06 | 5.01 |
|  |  |  | 5D | IWB42322 | Kukri_c20444_677 | [T/C] | 31,273,805 | ND | 0.08 | 0.06 | -7.24 | 6.10E-05 | 4.21 |
|  |  |  |  |  |  |  |  |  |  |  |  |  |  |

^a^Desirable SNP for reduced disease, based on the allele effect estimate, is in bold and underlined.

^b^Base pair (bp) location of the single base change in the IWGSC RefSeq v1.0.

^c^Consensus map position as reported in Wang et al. 2014, cM: centimorgans.

^d^MAF: minor allele frequency.

^e^The effect estimates the difference between the average phenotypic values of the homozygous A genotype relative to the homozygous B genotype.

NA: No associations detected at -log_10_(*p*) > 4.12.

ND: Not determined.
